# Statin Treatment Is Associated with Reduction in Serum Levels of Receptor Activator of NF-****κ****B Ligand and Neutrophil Activation in Patients with Severe Carotid Stenosis

**DOI:** 10.1155/2014/720987

**Published:** 2014-02-06

**Authors:** Sébastien Lenglet, Alessandra Quercioli, Mathias Fabre, Katia Galan, Graziano Pelli, Alessio Nencioni, Inga Bauer, Aldo Pende, Magaly Python, Maria Bertolotto, Giovanni Spinella, Bianca Pane, Domenico Palombo, Franco Dallegri, François Mach, Nicolas Vuilleumier, Fabrizio Montecucco

**Affiliations:** ^1^Division of Cardiology, Department of Internal Medicine, Foundation for Medical Researches, University of Geneva, 64 Avenue de la Roseraie, 1211 Geneva, Switzerland; ^2^Department of Internal Medicine, University of Genoa, 6 Viale Benedetto XV, 16132 Genoa, Italy; ^3^First Clinic of Internal Medicine, Department of Internal Medicine, University of Genoa School of Medicine, IRCCS Azienda Ospedaliera Universitaria San Martino-IST Istituto Nazionale per la Ricerca sul Cancro, Viale Benedetto XV, 16132 Genoa, Italy; ^4^Vascular and Endovascular Surgery Unit, Department of Surgery, San Martino Hospital, 10 Largo Rosanna Benzi, 16132 Genoa, Italy; ^5^Division of Laboratory Medicine, Department of Genetics and Laboratory Medicine, Geneva University Hospitals, 4 Rue Gabrielle-Perret-Gentil, 1205 Geneva, Switzerland

## Abstract

Systemic and intraplaque biomarkers have been widely investigated in clinical cohorts as promising surrogate parameters of cardiovascular vulnerability. In this pilot study, we investigated if systemic and intraplaque levels of calcification biomarkers were affected by treatment with a statin in a cohort of patients with severe carotid stenosis and being asymptomatic for ischemic stroke. Patients on statin therapy had reduced serum osteopontin (OPN), RANKL/osteoprotegerin (OPG) ratio, and MMP-9/pro-MMP-9 activity as compared to untreated patients. Statin-treated patients exhibited increased levels of collagen and reduced neutrophil infiltration in downstream portions of carotid plaques as compared to untreated controls. In upstream plaque portions, OPG content was increased in statin-treated patients as compared to controls. Other histological parameters (such as lipid, macrophage, smooth muscle cell, and MMP-9 content) as well as RANKL, RANK, and OPG mRNA levels did not differ between the two patient groups. Serum RANKL/OPG ratio positively correlated with serum levels of neutrophilic products, intraplaque neutrophil, and MMP-9 content within downstream portions of carotid plaques. In conclusion, statin treatment was associated with improvement in serum RANKL levels and reduced neutrophil activity both systemically and in atherosclerotic plaques.

## 1. Introduction

Atherosclerotic plaque calcification is considered a common process in advanced atherogenesis, although its association with increased plaque vulnerability remains unclear. On the one hand, the coronary calcium score (CAC) was shown to improve the Framingham risk score prediction in intermediate risk individuals [1] and to be an independent predictor of stroke in subjects deemed at low risk (accordingly to clinically based traditional risk stratifications tools) [2]. On the other hand, histological findings from human carotid atherosclerotic plaques demonstrated that symptomatic atheroma is characterized by reduced levels of calcification and increased inflammation as compared to asymptomatic plaques [3]. This scenario is further complicated by recent recommendations that “intraplaque vulnerability” is just considered as one of multiple determinants of cardiovascular risk (i.e., intraplaque, circulating, and peripheral tissue risk factors), suggesting the importance of defining systemic risk biomarkers and of understanding their pathophysiological role [4, 5]. Potential “systemic” biomarkers of intraplaque calcification include osteopontin (OPN), osteoprotegerin (OPG), and receptor activator of nuclear factor kB ligand (RANKL) [6–8]. In this context, we have recently shown that RANKL promotes the degranulation liberating proatherosclerotic products by neutrophils [9]. Notably, specific anti-RANKL neutralizing antibodies for clinical applications (i.e., denosumab) have been developed and are currently investigated as a way to block skeletal bone resorption in cancer diseases [10]. Since cardiovascular outcomes were not considered as endpoints in available cancer-related trials [11], the potential effects of those therapeutic compounds on atherogenesis are still elusive, and knowing whether conventional antiatherosclerotic treatments could affect RANKL signalling remains an open question.

Therefore, in this work, we studied the potential anti-RANKL effects of the 3-hydroxy-3-methylglutaryl-coenzyme A (HMG-CoA) reductase inhibitors (statins) in patients with severe carotid plaque stenosis and being asymptomatic for acute ischemic diseases.

Statins are the most common lipid-lowering drugs worldwide and are recommended for the prevention of cardiovascular diseases [12]. Their antiatherosclerotic effects include not only lipid profile improvements, but also additional “pleiotropic” activities (such as anti-inflammatory, antioxidative, and immunomodulatory activities), which all contribute to stabilizing atherosclerotic plaques [13, 14] and reduction of systemic atherosclerotic factors, such as C-reactive protein (CRP) [15]. For these reasons, statins might be very promising candidates to potentially reduce systemic and intraplaque RANKL levels and/or bioactivity (as a neutrophil activator) in advanced atherosclerosis.

## 2. Methods

### 2.1. Patients and Study Design

We conducted an observational cohort study between December 2010 and November 2011 at a single hospital (San Martino Hospital) in Genoa (Italy). Patients (*n* = 38) undergoing carotid endarterectomy (CEA) for extra cranial high-grade internal carotid stenosis (>70% luminal narrowing) [4–6] and being asymptomatic for ischemic stroke were included in the study. Patients were defined as asymptomatic when they had no history of ischemic symptoms and in the absence of signs of cerebral necrosis at magnetic resonance imaging (MRI) with diffusion sequences. The exclusion criteria were malignant hypertension, acute coronary artery disease, any cardiac arrhythmias, congestive heart failure (II, III, and IV NHYA classes), liver or renal disorders or function abnormalities, acute and chronic infectious disease, autoimmune and rheumatic diseases, osteoporosis, cancer, endocrine diseases, inflammatory bowel diseases and anti-inflammatory (other than aspirin) medications, oral anticoagulant treatments, hormone, and cytokine or growth factor therapies.

The day before endarterectomy, blood samples were obtained by peripheral venipuncture from these patients at fasting state to collect serum and plasma and to assess blood parameters. Medications reported in Table 1 were not modified in the 2 months prior to enrolment. Within the cohort, patients under treatment with a statin (*n* = 26) were compared with patients without statins (*n* = 12) for both systemic and intraplaque inflammatory and vascular parameters. Different statins and doses taken by patients were shown in Table 2. The Medical Ethics Committee of San Martino Hospital approved the study, and participants provided written informed consent. The study was conducted in compliance with the Declaration of Helsinki.

### 2.2. Power Estimation

Power calculation was based on prior published literature [16, 17]. Our sample size (26 patients under statins versus 12 patients without statin) allowed us to detect large effect sizes (>0.80) for systemic inflammatory markers or intraplaque parameters between symptomatic and asymptomatic patients with a power of 80%, taking a two-sided type I error of 5%.

### 2.3. Systemic Inflammatory Marker Detection

Serum C-reactive protein (CRP), osteopontin (OPN), osteoprotegerin (OPG), myeloperoxidase (MPO), matrix metalloproteinase (MMP)-8, MMP-9, tissue inhibitor of metalloproteinase (TIMP)-1, TIMP-2, TIMP-3, TIMP-4, and MMP9/TIMP-1 levels were measured by colorimetric enzyme-linked immunosorbent assay (ELISA, from R&D Systems, Minneapolis, Minnesota), following manufacturer's instructions. Serum RANKL levels were measured by ELISA (BioVendor GmbH, Heidelberg, Germany), following manufacturer's instructions. Serum neutrophil elastase levels were assessed by ELISA (Bender Med Systems GmbH, Vienna, Austria), following manufacturer's instructions. The limits of detection were 0.78 ng/mL for CRP, 0.312 ng/mL for OPN, 0.0625 ng/mL for OPG, 0.156 ng/mL for MPO, 0.156 ng/mL for MMP-8, 0.312 ng/mL for MMP-9, 0.156 ng/mL for TIMP-1, 0.156 ng/mL for TIMP-2, 0.0625 ng/mL for TIMP-3, 0.0781 ng/mL for TIMP-4, 0.0469 ng/mL for MMP/TIMP-1, 31.25 pg/mL for RANKL, and 0.156 ng/mL for neutrophil elastase. Mean intra- and interassay coefficients of variation (CV) were below 8% for all markers. Glucose, triglycerides, total cholesterol, low-density lipoprotein (LDL) cholesterol, and high-density lipoprotein (HDL) cholesterol were routinely measured and expressed in mg/dL. Serum insulin and C-peptide were routinely measured and expressed in mU/L and *μ*g/L, respectively.

### 2.4. Pro-MMP-9 Zymographic Assay

Pro-MMP-9 zymographic activity was assessed in human serum, as previously described [18]. 9% of SDS-polyacrylamide gels were copolymerized with gelatin (Sigma, St. Louis, MO). Equal amounts of patient serum (2 *μ*L, stored at −80°C) and 1 ng of recombinant pro-MMP-9 standard (Calbiochem, Lucerne, Switzerland) were loaded on gels in the absence of reducing agents. Then, gels were rinsed and stained with Coomassie Blue R-250. Zymographic results were expressed as pro-MMP-9 proteolytic activity and calculated on the basis of the following formula: Serum pro-MMP-9 = (*I*_obs_/*I*_std_) × *W*_std_, where *I*_obs_ and *I*_std_ are intensities of lytic areas produced in gels by samples and by standard pro-MMP-9 and *W*_std_ is the weight (1 ng) of standard pro-MMP-9 loaded onto the gel. Zymographic data were expressed as ng/mL of serum. Gelatinolytic bands were measured with a gel analysis system (GeneGenius, Syngene, Cambridge, UK).

### 2.5. Human Carotid Plaque Specimen Processing

Shortly after surgical excision, the internal carotid plaque specimens were taken from all patients and immediately transferred at 4°C to the laboratory for processing. Carotid plaques had the morphology of calcified advanced lesions with a lipid-rich acellular core. The internal carotid plaque specimens were cut perpendicularly to the long axis through the point of maximum stenosis to obtain two portions (upstream and downstream blood flow) [18]. Each portion was further divided perpendicularly to the long axis in the middle into two subsegments. One half was snap-frozen in liquid nitrogen and stored at −80°C for mRNA isolation, and the other half was frozen in cryoembedding medium for histological analysis.

### 2.6. Immunohistochemistry

Frozen upstream and downstream human carotid specimens were serially cut into eight 7 *μ*m sections per each portion separated by 105 *μ*m from each other [18]. Sections were fixed in acetone and immunostained with specific antibodies, anti-human smooth muscle actin (dilution: 1 : 100; Dako Corporation, Glostrup, Denmark), anti-human CD68 (dilution: 1 : 100; Dako Corporation), anti-human CD66b (dilution: 1 : 50; Beckman Coulter, Nyon, CH), anti-human matrix metalloproteinase (MMP)-9 (dilution: 1 : 250; Sothern Biotech, Birmingham, AL), anti-human OPG (dilution: 1 : 20; R&D Systems), and anti-human RANKL (dilution: 1 : 20; R&D Systems). Quantifications were performed with MetaMorph software. Results for these parameters were calculated and expressed as percentages of stained area on total lesion area or number of infiltrating cells on mm^2^ of lesion area.

### 2.7. Oil Red O Staining for Lipid Content

Eight sections per portion (upstream and downstream blood flow) of human carotid plaques were stained with Oil Red O, as previously described [18]. Sections were counterstained with Mayer's hemalum and rinsed in distilled water. Quantifications were performed with MetaMorph software. Data were calculated as ratios of stained area on total lesion area.

### 2.8. Sirius Red Staining for Collagen Content

Eight sections per portion (upstream and downstream blood flow) of human carotid plaques were rinsed with water and incubated with 0.1% Sirius red (Sigma Chemical Co, St Louis, MO) in saturated picric acid for 90 min. Sections were rinsed twice with 0.01 N HCl for 1 min and then immersed in water. After dehydration with ethanol for 30 seconds and cover-slipping, the sections were photographed with identical exposure settings under ordinary polychromatic or polarized light microscopy. Total collagen content was evaluated under polychromatic light. Interstitial collagen subtypes were evaluated using polarized light illumination; under this condition thicker type I collagen fibers appeared orange or red, whereas thinner type III collagen fibers were yellow or green [16]. Quantifications were performed with MetaMorph software. Data were calculated as percentages of stained area on total lesion area.

### 2.9. Real-Time RT-PCR

Total mRNA was isolated with Tri Reagent (MRC Inc.) from upstream or downstream specimens of human carotid plaques. Reverse transcription was performed using the ImProm-II Reverse Transcription System (Promega, Madison, WI) according to the manufacturer's instructions. Real-time PCR (StepOnePlus, Applied Biosystems) was performed with the ABsolute QPCR Mix (ABgene).

Specific primers and probes (Table 3) were used to determine the mRNA expression of RANK, RANKL, OPG, and RPS13 (housekeeping gene). The fold change of mRNA levels was calculated by the comparative *C*_*t*_ method. The measured *C*_*t*_ values were first normalized to the RPS13 internal control, by calculating delta *C*_*t*_ (Δ*C*_*t*_). This was achieved by subtracting the RPS13 *C*_*t*_ values from the gene of interest *C*_*t*_ value. Delta delta *C*_*t*_ (ΔΔ*C*_*t*_) was calculated by subtracting the designated baseline control group Δ*C*_*t*_ value from the study group Δ*C*_*t*_ values. The ΔΔ*C*_*t*_ was then plotted as a relative fold change with the following formula: 2^−ΔΔ*C*_*t*_^.

### 2.10. Statistical Analysis

Patient characteristics were described one day before endarterectomy. Patients asymptomatic for ischemic stroke and on statin treatment were compared to patients without statins using the Pearson's chi-square test or the Fisher's exact test (when appropriate) for the comparison of qualitative variables. The Mann-Whitney nonparametric test was used for comparisons of continuous variables. Results were expressed as medians (interquartile range (IQR)). Spearman's rank correlation coefficients were used to assess correlations between serum RANKL/OPG ratio and levels of serum neutrophil products and intraplaque parameters in both upstream and downstream regions of carotid atherosclerotic plaques, respectively. Values of *P* < 0.05 (two-tailed) were considered significant. All analyses were done with GraphPad Instat software version 3.05 (GraphPad Software).

## 3. Results

### 3.1. Baseline Population Characteristics

Clinical features, laboratory parameters, and medications of our cohort of asymptomatic patients with severe carotid stenosis (*n* = 38) are shown in Table 1. There was no significant difference between patients under or without treatment with a statin in terms of age, sex, classical cardiovascular risk factors, hematological parameters, and medications except for a slight but significant (*P* = 0.0429) decrease on circulating monocyte count in statin-treated group. Table 2 summarizes the statins used by the patients and their dosage. Only treatments with low doses of statins [19] were investigated in the present study (Table 2).

### 3.2. Statin Treatment Is Associated with the Modulation of Serum Levels of Calcification Biomarkers and MMP-9

The first goal of this study was to determine if statin treatment was associated with improvements in the systemic levels of cardiovascular risk factors, calcification biomarkers, and neutrophil degranulation products, with CRP being the first biomarker to be analyzed. Statin-treated patients had similar CRP serum levels as compared to statin-untreated controls (Table 4). With respect to calcification biomarkers, serum levels of OPN and RANKL were significantly reduced, while OPG was increased in statin-treated patients as compared to patients without statins. In line with the observed changes in RANKL and OPG levels, the RANKL/OPG ratio (indicating the circulating RANKL free fraction) was significantly decreased in statin-treated patients as compared to controls. With respect to neutrophil degranulation products, we did not find any difference between the two patient groups in terms of serum MPO, neutrophil elastase, and MMP-8 levels and of protective tissue inhibitor of metalloproteinases (TIMP-1, -2, -3, and -4) (Table 4). However, the MMP-9 level and pro-MMP-9 activity were significantly reduced in patients receiving statin therapy as compared to those without statins (Table 4).

### 3.3. Statin Treatment Is Associated with Reduction in Neutrophil Infiltrates and Vulnerability in Carotid Plaque Portions Downstream Blood Flow

**Figure 1:**
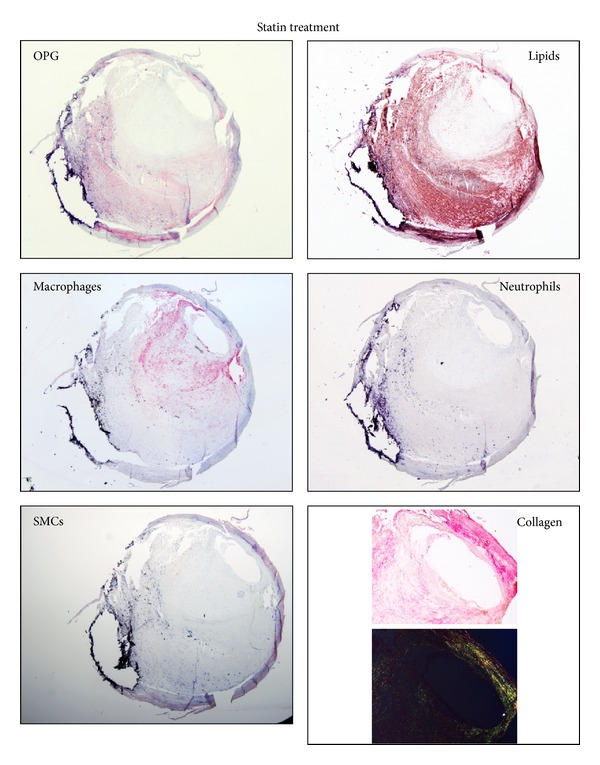
Representative microphotographs of consecutive cryosections from upstream regions of carotid plaques from patients on statin treatment. Staining for osteoprotegerin (OPG), lipids, macrophages, neutrophils and smooth muscle cells (SMCs), and collagen is shown.

**Figure 2:**
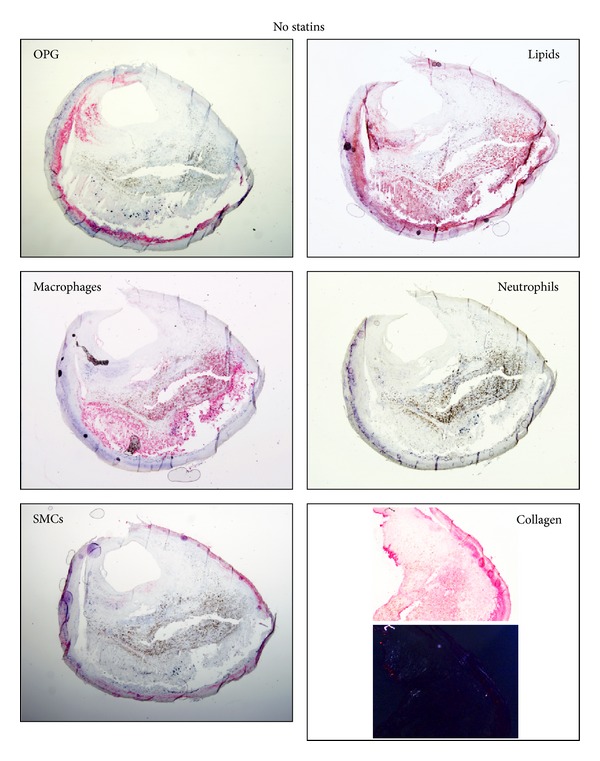
Representative microphotographs of consecutive cryosections from upstream regions of carotid plaques from patients without statins. Staining for osteoprotegerin (OPG), lipids, macrophages, neutrophils and smooth muscle cells (SMCs), and collagen is shown.

Taking into account the elevated heterogeneity of human carotid plaques [17, 18, 20], the effect of statin treatment on intraplaque inflammatory patterns and calcification biomarkers was assessed in different regions (upstream and downstream blood flow, resp.). In upstream portions, no differences were found between the two patient groups in terms of lipid and collagen content and of cell composition (SMCs, macrophages, neutrophils, and MMP-9) (Table 5, Figures 1 and 2). In downstream regions, the total collagen and collagen I contents were significantly higher in the statin-treated group as compared to the group with no statin use. Notably, this effect was associated with a virtual abrogation of intraplaque neutrophil infiltration in patients treated with statins as compared to untreated patients. Despite being close to significance (*P* = 0.0574), a similar trend in intraplaque content of the neutrophilic product MMP-9 was also observed (Table 5). No difference between the two patient groups was found in terms of intraplaque lipids and of infiltration by other cell subsets, such as SMCs and macrophages (Table 5).

Calcification biomarkers have previously been reported to be expressed within atherosclerotic plaques [21]. Thus, we focused on the expression of these markers in our cohort of patients both at mRNA and protein level. RANKL was almost undetectable in both groups at both mRNA (upstream plaques: detectable in 14 statin and 10 no statin; downstream plaques: in 17 statin and 11 no statin) and protein levels (detectable in 4 downstream portions of carotid plaques from statin-treated patients, data not shown). Statin treatment was not associated with an altered expression of RANKL, RANK (RANKL's receptor), and OPG mRNA as compared to control patients without statins (Table 5). At the protein level, intraplaque OPG expression was weak in both groups (Table 5). A significant increase in OPG protein levels was shown in upstream regions of carotid plaques from statin-treated versus untreated patients, whereas no statistically significant difference in downstream plaque portions could be detected (Table 5). Finally, the expression pattern of OPG in carotid plaques suggested that this protein did not colocalize with macrophages or neutrophils but rather with intimal SMCs and lipids (Figures 1 and 2). This result was similar in both patients' groups (Figures 1 and 2).

### 3.4. RANKL/OPG Ratio Positively Correlates with Neutrophil Degranulation Products Both in the Systemic Circulation and within Carotid Plaques

Spearman rank correlations were performed in order to identify potential associations between free serum RANKL levels (RANKL/OPG ratio) on the one hand and systemic and intraplaque neutrophil products on the other in our two groups of patients (statin treatment versus no statin treatment). RANKL/OPG ratio was found to positively correlate with serum CRP, MPO, and neutrophil elastase levels, as well as with serum pro-MMP-9 activity (Table 6). Though not statistically significant (*P* = 0.0614), a positive association between RANKL/OPG ratio and MMP-9 serum levels was observed (Table 6). No significant association between RANKL/OPG ratio and serum MMP-8 was detected (Table 6). Serum RANKL/OPG ratio was also shown to inversely correlate with intraplaque lipid content in upstream regions of carotid plaques and to positively correlate with neutrophil and MMP-9 content in downstream portions. No additional significant correlations were shown for other intraplaque parameters, such as collagen content, SMCs, and macrophages, in the different intraplaque regions (Table 6).

## 4. Discussion

The main result of this study is represented by the finding that an ongoing treatment with statins is associated with alterations in serum levels of biomarkers of plaque calcification such as OPN, RANKL, and OPG in asymptomatic patients with a severe carotid stenosis undergoing a carotid endarterectomy. In particular, the free circulating fraction of RANKL (not bound to OPG, expressed as RANKL/OPG ratio) was strongly reduced in statin-treated patients as compared to controls without a statin. Conversely, we did not detect any relevant change in intraplaque RANKL/OPG levels between statin-treated and -untreated patients. Notably, we were only able to detect very low intraplaque RANKL levels, suggesting that this mediator may be preferentially expressed in the blood stream, instead of within atherosclerotic plaques [9]. Our data also suggest a new, potential, beneficial effect of statins as they may directly reduce the levels of circulating free RANKL, thereby preventing its detrimental effects on proatherosclerotic cell subsets (including neutrophils). A potential reduction in both OPG and RANKL serum levels after atorvastatin treatment was previously shown by Dimitrow and coworkers in patients with aortic sclerosis or mild aortic stenosis [22]. In this paper, we confirmed that statin treatment was associated with a reduction in RANKL serum levels, but, surprisingly, we showed an increase in serum OPG (the decoy receptor of soluble RANKL that blocks its bioactivity). The role of OPG in cardiovascular diseases requires further clarifications, since its levels have been associated with cardiovascular risk without reporting a clear proatherosclerotic direct bioactivity of this molecule [23–25]. In OPG^−/−^ mice, treatment with recombinant OPG induced signs of fibrosis, promoted intraplaque SMCs accumulation, collagen fiber formation, and development of fibrous caps, thus supporting a pathogenic role of OPG in the development, progression, and instability of atherosclerotic lesions [26]. However, additional studies that should be aimed, for instance, at identifying OPG serum cutoff values, are needed to clarify the exact nature of OPG as an innocent bystander or an active player in cardiovascular diseases.

The lack of efficacy of statins in the modulation of intraplaque RANKL/OPG levels might be also explained by different findings. First of all, all statins were administered to the patients from our cohort at a low dose [19]. Thus, their tissue concentration may have been not high enough to reduce intraplaque inflammation. To some extent, this hypothesis is corroborated by the weak improvements in plaque stability parameters that we found in patients on statin as compared to untreated controls. Namely, we only detected a small reduction in neutrophil and MMP-9 content and an increase in intraplaque collagen in the downstream portions of carotid plaques in statin-treated patients versus controls, while Crisby and colleagues [16] had previously found that pravastatin treatment markedly improved carotid plaque stability as compared with control patients by reducing macrophages, T cells, and MMP-2 and by increasing protective intraplaque collagen content.

Another possible reason for our inability to detect a modulation of intraplaque RANKL/OPG levels is that our approach investigated atherosclerotic plaques as very heterogeneous tissues (as a result of different types of shear stress exposure) [20], while Crisby and colleagues evaluated the plaque a single homogeneous tissue. Thus, potential differences from the study may reflect the different approach.

Finally, the fact that different statins (rosuvastatin, simvastatin, and atorvastatin) were administrated to the patients in our study might also explain, at least in part, the limited activity of statins on intraplaque parameters that we observed. A similar result was also reported by Verhoeven and coworkers [27], who found that macrophage content might be particularly affected by atorvastatin treatment but not by other statins.

Notably, in out cohort of patients, treatment with statins did not reduce plaque lipid-rich core size, which is a key vulnerability marker in atherosclerotic plaques. This result may be surprising since Crisby and colleagues [16] clearly demonstrated that the plaques of pravastatin-treated patients with symptomatic carotid artery stenosis had substantially less lipid (3 times less) as compared to control subjects. On the other hand, our findings are supported by a more recent study on coronary atherosclerosis, in which the authors demonstrated that statins significantly decreased plaque size only if LDL-c was <100 mg/mL [28]. In our study, we did not observe such important reduction in LDL-c levels in patients treated with statins (median value 104 mg/dL and LDL-c levels < 100 mg/dL in only 12 patients (46.2%)). Therefore, taking all of these aspects into account and in accordance with the current international recommendations [12], our study suggests that the use of high-dose and long-term lipid-lowering strategy with a statin has to be considered when the goal is to reduce intraplaque vulnerability and cardiovascular adverse events in patients with advanced atherosclerosis [29, 30].

Another important result of this study is represented by the confirmation of serum direct correlations between free RANKL levels and neutrophil products. A significant positive association between levels of RANKL/OPG ratio and other neutrophil products (such as MPO and neutrophil elastase) was also demonstrated, confirming a strong induction of neutrophil degranulation mediated by this cytokine [9]. This robust activation of neutrophils (positively associated in our study with serum RANKL levels) was particularly observed in the systemic circulation instead of within the carotid plaque. Confirming a previous study showing an active role of RANKL in circulating neutrophil degranulation of proatherosclerotic products (such as MMP-9) in patients with increased CAC and *in vitro* [9], this study further confirmed these cells as a major source of proteinases favoring systemic atherosclerotic vulnerability.

This pilot explanatory study has several imitations. Firstly, the number of participants is relatively small (also with a disproportion between statin-treated patients (*n* = 26) and untreated controls (*n* = 12)), thus preventing the detection of small differences in all markers between the two groups. Secondly, as we have mentioned in the discussion, we do not know how different lipid-lowering regimens (i.e., different statins and high-dose versus low-dose therapeutic approaches) can modulate the inflammatory burden within serum and atherosclerotic plaques. Anyway, our results might unveil a novel “pleiotropic” activity for statins selectively targeting RANKL/OPG system and potentially neutrophil activation and plaque calcification. In conclusion, we demonstrated that patients treated with a statin (low-dose) for severe carotid stenosis had reduced serum levels of free RANKL (RANKL/OPG ratio) as compared to controls without statins. This reduction of RANKL levels was positively associated with a concomitant reduction in neutrophil degranulation products, primarily in the systemic circulation and also within carotid plaques. This hypothesis-generating study may suggest that treatments that more selectively target the RANKL/OPG system might be beneficial in advanced atherosclerosis to reduce neutrophil-mediated patient vulnerability.

## Figures and Tables

**Table 1:** Clinical characteristics and medications of the study population.

Characteristics	Statin (*n* = 26)	No statin (*n* = 12)	*P* value
Age, yr (IQR)	72.0 (66.0–77.3)	71.5 (67.3–76.7)	0.9249
Males, *n* (%)	14 (53.8)	7 (58.3)	1.0000
CV risk factors			
Blood pressure, mmHg			
Systolic (IQR)	130.0 (125.0–141.3)	130.0 (125.0–140.0)	0.6826
Diastolic (IQR)	85.0 (80.0–90.0)	80.5 (80.0–84.5)	0.4686
Waist Circumference, cm (IQR)	91.0 (88.0–98.0)	91.5 (90.0–95.0)	0.7414
Current smoking, *n* (%)	7 (26.9)	3 (25.0)	1.0000
Type 2 diabetes, *n* (%)	11 (42.3)	3 (25.0)	0.4722
Dislipidemia, *n* (%)	16 (61.5)	4 (33.3)	0.1643
Hypertension, *n* (%)	21 (80.8)	10 (83.3)	1.0000
Chronic CAD*, *n* (%)	7 (26.9)	3 (25)	1.0000
Carotid stenosis % lumen (IQR)	75 (70–80)	80 (70–85)	0.2349
Biological parameters			
Total WBC^†^, number ×10^9^/L (IQR)	6.35 (5.53–7.43)	6.56 (5.60–8.98)	0.4053
Neutrophils, number ×10^9^/L (IQR)	3.90 (3.38–5.05)	4.36 (3.57–6.14)	0.4996
Lymphocytes, number ×10^9^/L (IQR)	1.58 (1.24–1.96)	1.56 (1.12–1.84)	0.6716
Monocytes, number ×10^9^/L (IQR)	0.37 (0.30–0.49)	0.44 (0.38–0.53)	0.0429
Red blood cells, number ×10^12^/L (IQR)	4.60 (4.10–4.85)	4.65 (4.53–5.00)	0.2513
Platelet, number ×10^9^/L (IQR)	207.5 (178.8–238.8)	188.5 (175.5–219.8)	0.5096
Plasma fibrinogen, g/L (IQR)	3.65 (3.25–4.79)	4.09 (3.69–5.11)	0.3146
Serum total-c^‡^, mg/dl (IQR)	189.0 (164.0–215.0)	202.0 (159.3–209.3)	0.6150
Serum LDL-c^§^, mg/dl (IQR)	104.0 (81.2–130.0)	118.8 (77.0–132.2)	0.6380
Serum HDL-c^||^, mg/dl (IQR)	60.0 (41.5–69.0)	49.5 (41.5–64.0)	0.4754
Serum triglycerides, mg/dl (IQR)	124.0 (83.5–160.0)	85.0 (68.0–118.8)	0.2237
Serum glycaemia, mg/dl (IQR)	101.0 (96.5–153.0)	111.0 (95.8–124.0)	0.6497
Serum insulinemia, mU/L (IQR)	7.5 (5.8–16.3)	5.6 (3.7–12.2)	0.0747
Serum C-peptidemia, *μ*g/L (IQR)	2.35 (1.92–3.97)	2.06 (1.60–2.61)	0.1894
Antiplatelets, *n* (%)	23 (88.5)	10 (83.3)	0.6426
Diuretics, *n* (%)	2 (7.7)	4 (33.3)	0.0661
ACE^#^ inhibitors, *n* (%)	2 (7.7)	1 (8.3)	0.2295
ARBs**, *n* (%)	11 (42.3)	6 (50.0)	0.7342
Beta-blockers, *n* (%)	10 (38.5)	3 (25.0)	0.4859
Calcium channel blockers, *n* (%)	9 (34.6)	4 (33.3)	1.0000
Oral antidiabetics, *n* (%)	7 (26.9)	3 (25.0)	1.0000

Continuous variables are expressed as median (interquartile range (IQR)).

*CAD: coronary artery disease.

^†^WBC: white blood cells.

^‡^total-c: total cholesterol.

^§^LDL-c: low-density lipoprotein cholesterol.

^||^HDL-c: high-density lipoprotein cholesterol.

^#^ACE: angiotensin converting enzyme.

**ARBs: angiotensin receptor blockers.

**Table 2:** Different statin treatments.

Statin (dose)	*n* (%)
Rosuvastatin (5 mg/day)	12 (46.15)
Simvastatin (40 mg/day)	6 (23.08)
Atorvastatin (20 mg/day)	8 (30.77)

**Table 3:** Human primers used for real-time PCR.

Gene	Function	Nucleotide sequence	Size (bp)	Accession number
RPS13	Fw	5′-CGTCCCCACTTGGTTGAAG-3′	90	NM_001017
	Rv	5′-CCGATCTGTGAAGGAGTAAGG-3′		
	Probe	5′-ACATCTGACGACGTGAAGGAGCAGATT-3′		

TNFSF11 (RANKL)	Fw	5′-AAGGAGCTGTGCAAAAGGAA-3′	75	NM_003701
	Rv	5′-CATCCACCATCGCTTTCTCT-3′		
	Probe	5′-CGTTGGATCACAGCACATCAGAGC-3′		

TNFRSF11A (RANK)	Fw	5′-CAGCTAATTTGTGGCACTGG-3′	68	NM_003839
	Rv	5′-ACCTGAGGACTCCTTATCTCCA-3′		
	Probe	5′-CAATGAGGCTTGTGGCCGCCTA-3′		

TNFRSF11B (OPG)	Fw	5′-TGGAATAGATGTTACCCTGTGTG-3′	149	NM_002546
	Rv	5′-TGTGTTGCCGTTTTATCCTCT-3′		
	Probe	5′-AGGCATTCTTCAGGTTTGCTGTTCC-3′		

**Table 4:** Serum cardiovascular risk markers.

Characteristics	Statin (*n* = 26)	No statin (*n* = 12)	*P*-value
CRP*, mg/L	2.4 (0.96–4.03)	4.1 (1.43–8.41)	0.1782
OPN, ng/mL	17.5 (12.8–23.8)	29.4 (23.2–34.6)	0.0131
RANKL, pg/mL	1022 (247–1669)	1839 (1551–2803)	0.0050
OPG, pg/mL	167.8 (62.3–318.1)	62.3 (62.3–432.0)	0.0182
RANKL/OPG ratio	5.8 (1.5–17.1)	27.2 (15.5–43.3)	0.0004
MPO, ng/mL	265.2 (101.8–531.6)	466.0 (313.7–841.9)	0.1014
Neutrophil elastase, ng/mL	211.1 (109.1–318.6)	325.3 (143.1–450.2)	0.1489
MMP^†^-8, ng/mL	16.5 (5.8–23.7)	12.7 (4.0–24.7)	0.7579
MMP-9, ng/mL	231.5 (134.9–431.6)	479.0 (352.8–812.5)	0.0461
Pro-MMP-9 activity, ng/mL	6.8 (3.1–14.4)	13.1 (11.9–18.2)	0.0310
MMP9/TIMP-1, ng/mL	10.6 (6.4–18.4)	14.4 (2.8–23.0)	0.8839
TIMP^‡^-1, ng/mL	191.3 (120.0–216.7)	211.0 (147.6–264.6)	0.3389
TIMP-2, ng/mL	101.4 (65.4–124.5)	97.0 (73.0–112.2)	0.8078
TIMP-3, ng/mL	8.1 (4.7–14.9)	12.5 (6.2–13.8)	0.5824
TIMP-4, ng/mL	5.4 (3.6–6.6)	4.2 (4.0–6.1)	0.7579

Data are expressed as median (interquartile range (IQR)).

*CRP: C-reactive protein.

^†^MMP: matrix metalloproteinase.

^‡^TIMP: tissue inhibitor of metalloproteinase.

**Table 5:** Parameters of intraplaque vulnerability.

Characteristics	Statin (*n* = 26)	No statin (*n* = 12)	*P*-value
Upstream portion			
% of lipid	4.63 (1.84–8.63)	4.36 (3.17–5.54)	0.6524
% of total collagen	41.24 (18.91–52.70)	30.43 (21.25–43.09)	0.6786
% of collagen I	8.97 (4.27–15.98)	6.42 (4.71–10.04)	0.4385
% of collagen III	21.79 (15.32–37.75)	20.33 (16.67–31.94)	0.9569
% of smooth muscle cell-rich area	8.85 (3.75–19.57)	8.00 (3.22–14.48)	0.6013
% of macrophage-rich area	2.96 (0.88–9.06)	2.08 (0.27–6.69)	0.4823
Neutrophils/mm^2^	0.75 (0.52–1.32)	0.81 (0.30–12.47)	0.6786
% of MMP*-9	0.71 (0.33–1.36)	0.39 (0.21–7.19)	0.6524
RANK^†^ mRNA, fold increase	0.49 (0.33–1.15)	0.81 (0.31–1.74)	0.5725
RANKL^‡^ mRNA, fold increase	0.78 (0.30–1.24)	0.61 (0.32–0.98)	0.8859
OPG^§^ mRNA, fold increase	0.84 (0.57–1.47)	0.83 (0.50–1.17)	0.7944
% of OPG	2.71 (1.77–6.06)	0.50 (0.10–2.34)	0.0150

Downstream portion			
% of lipid	4.39 (2.83–8.69)	4.94 (2.13–6.63)	0.4884
% of total collagen	18.45 (2.07–45.87)	5.06 (0.50–10.28)	0.0289
% of collagen I	5.56 (0.67–20.74)	0.67 (0.11–3.37)	0.0345
% of collagen III	10.61 (2.15–25.11)	1.98 (0.70–8.57)	0.0728
% of smooth muscle cell-rich area	3.87 (1.50–5.15)	3.59 (2.42–4.30)	0.8173
% of macrophage-rich area	1.97 (1.02–8.29)	9.00 (2.24–11.70)	0.1060
Neutrophils/mm^2^	0.26 (0.15–0.93)	3.77 (1.20–8.16)	0.0001
% of MMP-9	4.05 (0.68–12.30)	10.12 (3.86–13.81)	0.0574
RANK mRNA, fold increase	0.99 (0.48–2.77)	0.62 (0.38–2.43)	0.4601
RANKL mRNA, fold increase	0.84 (0.45–1.25)	0.59 (0.17–1.60)	0.5165
OPG mRNA, fold increase	1.38 (1.06–1.71)	1.06 (0.80–1.60)	0.2117
% of OPG	0.27 (0.01–0.96)	0.28 (0.02–0.67)	0.7746

Data are expressed as median (interquartile range (IQR)).

*MMP: matrix metalloproteinase.

^†^RANK: receptor activator of nuclear factor-*κ*B.

^‡^RANKL: receptor activator of nuclear factor-*κ*B ligand.

^§^OPG: osteoprotegerin.

**Table 6:** Spearman rank correlation between serum RANKL/OPG ratio and systemic and intraplaque parameters (upstream or downstream regions) of vulnerability.

	Spearman's correlation coefficient (*r*)	*P*-value
Serum RANKL/OPG ratio		
Systemic		
CRP, mg/L	0.3549	0.0311
MPO, ng/mL	0.6036	0.0001
Neutrophil elastase, ng/mL	0.5223	0.0009
MMP-8, ng/mL	0.0436	0.7977
MMP-9, ng/mL	0.3106	0.0614
Pro-MMP-9 activity, ng/mL	0.3248	0.0498
Intraplaque upstream		
versus lipids	−0.3456	0.0489
versus total collagen	0.0077	0.9661
versus collagen I	0.0879	0.6267
versus collagen III	0.0906	0.6162
versus SMCs	−0.0832	0.6452
versus macrophages	−0.0552	0.7605
versus neutrophils	0.1180	0.5132
versus MMP-9	−0.2647	0.1366
Intraplaque downstream		
versus lipids	−0.0946	0.5947
versus total collagen	−0.1383	0.4355
versus collagen I	−0.1636	0.3551
versus collagen III	−0.0805	0.6508
versus SMCs	−0.0118	0.9473
versus macrophages	0.2461	0.1605
versus neutrophils	0.3756	0.0285
versus MMP-9	0.3510	0.0418
